# A feasibility randomised controlled trial of short-term fasting prior to CAPOX chemotherapy for stage 2/3 colorectal cancer: SWiFT protocol

**DOI:** 10.1186/s40814-019-0505-7

**Published:** 2019-11-20

**Authors:** Ellie Shingler, Claire Perks, Georgia Herbert, Andy Ness, Charlotte Atkinson

**Affiliations:** 1 NIHR Bristol Biomedical Research Centre (Nutrition Theme), Level 3, University Hospitals Bristol Education Centre, Upper Maudlin Street, Bristol, BS2 8AE England; 2 School of Clinical Sciences, University of Bristol, Bristol, England

**Keywords:** Short-term fast, Dietary restriction, Colorectal cancer, Feasibility, RCT

## Abstract

**Background:**

Capecitabine and oxaliplatin (CAPOX) chemotherapy is a standard treatment for stage 2/3 colorectal cancer. Treatment is associated with dose-limiting toxicities such as neutropenia, vomiting, diarrhoea, and stomatitis. Short-term fasting prior to chemotherapy may help protect normal cells from the toxic effects of chemotherapy by allowing them to conserve energy for maintenance and repair. However, there is a lack of evidence to support the efficacy of short-term fasting in protecting against chemotherapy-related toxicities in humans, and it is not known whether people due to undergo chemotherapy will be willing and able to follow a short-term fast. Preliminary data confirming this is feasible are required before adequately powered trials can be designed and conducted.

**Methods:**

The short-term, water only, fasting trial (SWiFT) is a two-armed feasibility randomised controlled trial, aiming to recruit 30 people scheduled to begin routine treatment with CAPOX chemotherapy for stage 2/3 colorectal cancer. Participants will be randomly allocated, in a 1:1 ratio, to either a 36-h fast or standard dietary advice prior to chemotherapy administration for the first 3 cycles of chemotherapy. The primary outcome measures will assess the feasibility of the trial and include: adherence to intervention, recruitment, retention, and data completion rates as well as the acceptability of the intervention which will be qualitatively assessed. The secondary outcome measures aim to provide further information on possible outcomes of interest for a definitive trial and include side effects of chemotherapy, quality of life, markers of cellular metabolism and inflammation, appetite, and sarcopenia.

**Discussion:**

It is not known whether it is possible to recruit to a trial of short-term fasting in this population, or whether participants would be able to adhere to the intervention. Therefore, we aim to test the feasibility of a pre-chemotherapy, 36-h, water-only fast in people receiving CAPOX chemotherapy for stage 2/3 colorectal cancer.

**Trial registration:**

This trial has been registered with the ISRCTN Registry. Trial registration no: ISRCTN17994717. Date of registration: 23 October 2018. URL: http://www.isrctn.com/ISRCTN17994717

## Background

During times of nutrient scarcity, normally functioning body cells have the ability to switch from a state of growth and development to a state of maintenance and repair, which enables cells to conserve energy [1, 2]. This process is mediated in part by a reduction in glucose, insulin, and IGF-1 (Insulin-like growth factor-1) which in turn alters cellular pathways, reducing activation of those involved in cell proliferation and growt h[2, 3].

Conversely, as cancer is a disease of uncontrolled cell growth, genetic mutations in tumour cells enable them to continue to proliferate, even when nutrients are scarc e[4]. This is due to loss-of-function mutations in tumour-suppressor genes (Rb, p53, and PTEN) which lead to insensitivity to growth-inhibitory signals. In addition, mutations in oncogenes (Ras, Akt, and mTOR) activate proliferation pathways, even in the absence of growth signal s[3].

This difference between healthy and tumour cells in response to food restriction has been coined “Differential Stress Resistance (DSR)”. Pre-clinical research in animal models suggests that DSR could be exploited during chemotherapy to limit the toxic effects of chemotherapy in healthy cells, while leaving tumour cells susceptible to treatment [5].

The exact mechanisms behind DSR are not fully understood. It is believed they may be partially mediated by the reduction in blood glucose and growth factors such as Insulin-like Growth Factors (IGFs) brought about by fasting. Within healthy cells, this negatively regulates downstream cellular pathways such as the Ras/MAPK and P13K/Akt pathways which are promoters of cellular proliferation [3]. This allows the cells to cease growth and convert energy to cell maintenance.

Another mechanism through which DSR may protect healthy cells is through increased autophagy. Calorie restriction activates the enzyme “AMP activated protein kinase” which increases autophagy. Autophagy can target defective organelles to be degraded into substrate for use in energy production and repai r[3]. Cancer is often associated with a defect in autophagic capacity as oncogenes Akt and P13k inhibit autophagy while the tumour suppressor gene PTEN, which loses function in tumour cells, would usually upregulate it. The decrease in growth factors associated with fasting may therefore lead to the upregulation of autophagy in normal cells but not in cancer cells, allowing them to degrade organelles for energy us e[6].

Although some studies of fasting during chemotherapy in cell lines and animal models have shown promising results, it is unclear how well these findings translate to human s[3, 7, 8].

To date, two early-stage trials on short-term fasting during chemotherapy have been completed: A pilot trial in 13 people with breast cance r[9] and a dose-escalating safety and feasibility trial in 20 people receiving platinum-based chemotherapy for any cancer typ e[10]. These studies show fasting up to 72hrs around the time of chemotherapy administration to be safe and feasible; however, the 72hour fast may have been subject to poorer adherence. There was some evidence of reduced haematologic toxicities between groups, but limited evidence of differences between groups for other chemotherapy side effects. However, these trials were not powered to provide evidence of differences in toxicities between groups, so the results should be treated as preliminary findings that require replication in adequately-powered studies.

No trials have been conducted to date in people receiving chemotherapy for colorectal cancer. A standard treatment in the UK for stage 3 colorectal cancer (or stage 2 colorectal cancer that is at high risk of local recurrence) is surgical resection, followed by Capecitabine and Oxaliplatin (CAPOX) chemotherapy [11]. Dose-limiting toxicities for this combined regimen include vomiting, diarrhoea, and stomatitis [12]. When used within an RCT, withdrawal rates on CAPOX due to adverse events were 21% [13]. Therefore, interventions which aim to limit toxicities in this treatment group are of interest. This identifies colorectal cancer as an understudied cancer site in terms of fasting to induce DSR as a means of reducing chemotherapy toxicities.

## Methods

### Trial aim and design

Patient and public involvement were used to inform the design of SWiFT. It is a two-armed randomised controlled feasibility trial. 30 people scheduled to begin routine treatment with CAPOX chemotherapy for stage 2/3 colorectal cancer will be randomly allocated, in a 1:1 ratio, to either a 36-hour fast or standard dietary advice, to be implemented prior to each of the first three treatment cycles.

The aim of the study is to determine whether a trial of a 36-hour pre-chemotherapy fast is feasible in people receiving CAPOX chemotherapy for stage 2/3 colorectal cancer.

### Outcome measures

The primary outcome measures assess the feasibility of this trial. These are

#### Recruitment rates

Calculated as the percentage of eligible patients recruited each month, as recorded in the recruitment logs at each site.

#### Adherence to intervention

Assessed by analysis of self-reported food logs, completed by participants during the 36-h fast. Participants will be considered to have adhered to the fast if they consume less than 14% of their basal metabolic rate (BMR) requirements (kcal/day calculated using the Oxford equations for BMR [14]), in the 36 h prior to chemotherapy administration. This equates to approximately 200 kcal per day, depending on age and gender. The aim of using a cut-off of 14% BMR is to allow participants to consume small amounts of food if they need to mitigate any side effects of fasting, while keeping the participant in the metabolically altered state associated with fasting. This cut-off has been used in previous trials of fasting [10]. To encourage participants to only consume a small number of calories, a list of 50 kcal snacks will be provided. The percentage of adherent participants will be reported for each cycle. Reasons for non-adherence will also be recorded.

#### Retention rates

Calculated as the number of participants who completed data collection for each fasting cycle (both self-reported data and clinical care team recorded data associated with that cycle) divided by the number of participants randomised.

#### Acceptability and tolerability of the intervention

Qualitatively assessed through in-depth semi-structured interviews with trial participants.

#### Data completion rates

Completeness of data will be assessed for all measures at each cycle. The secondary outcome measures aim to provide further information on possible outcomes of interest in a definitive trial. Including these in the feasibility trial will allow for the data collection methods to be assessed as well as providing information on the variance of each outcome. The impact of fasting on these measures could then be tested in a suitably powered trial. The secondary outcome measures are:

#### Side effects of chemotherapy

Measured using the Patient-Reported Outcomes version of the Common Terminology Criteria for Adverse Events (PRO-CTCAE™) [15], Full-blood count (FBC) and blood chemistry analysis. Short-term dietary fasting may result in differential stress resistance between normal and tumour cells, which may render tumour cells more susceptible to chemotherapy than normal cells. Patient-reported side effects will be collected on day 1 of each cycle (prior to chemotherapy administration) and at home on days 3 and 7 (via participant-completed questionnaires) to capture the transient nature of side effects. Data will also be recorded on whether participants completed their first 3 cycles of chemotherapy and reasons for dose reductions/delays/early termination if they occurred.

#### Quality of Life

Measured using the EQ-5D-5L health-related quality of life instrument [16]. This would be used to explore whether fasting or its impact on chemotherapy side effects, increases quality of life (QoL).

#### Haematologic toxicities

Assessed using routine FBC data collected prior to each round of chemotherapy and classified according to CTCAE criteria [17].

#### Markers of cellular metabolism

Measures will include glucose, insulin, IGF-I, IGF-II, IGF-binding protein (IGFBP)-2 and IGFBP-3. These will be used to study the effect of the fast on markers of cellular metabolism. They will also be considered in conjunction with self-reported dietary intake to explore adherence to the intervention, as the level of these markers would be expected to be reduced in adherent participants. Baseline samples will be collected prior to fasting when participants attend for routine pre-chemotherapy blood tests (approx. 4 days prior to cycle 1). Follow-up samples will be collected immediately prior to chemotherapy administration at cycles 1 and 3.

#### Markers of inflammation

C-reactive protein (CRP) will be measured at baseline (pre-fast) and prior to chemotherapy administration at cycles 1 and 3. In a full-powered trial, this would be used to explore whether fasting reduces inflammation.

#### Appetite

Self-reported on visual analogue scales [18]. As chemotherapy can alter taste and appetite [19], measuring appetite is of interest to explore whether fasting negates reduced appetite through decreased treatment side effects.

#### Sarcopenia

Assessed using computerised tomography (CT) and handgrip dynamometer [20]. Single axial images of the third lumbar (L3) level muscle mass, taken from pre-chemotherapy staging and follow-up CT scans conducted as part of routine care, will be analysed for body composition using SliceOMatic software [21]. Handgrip strength will be measured three times in the dominant hand, while the participant is in a seated position, arms supported at right angles, and feet on the floor. Measurements will be taken by the research site’s clinical trial officer/research nurse who will be trained in following the trial’s standard operating procedure for measuring handgrip strength. The mean of the three measures will be used to assess handgrip strength, using cut-off values defined by the European Working Group on Sarcopenia in Older People (EWGSOP) to identify low grip strength [22]. These measures will allow us to inform future trials on the prevalence of sarcopenia in this population and explore the safety of fasting in relation to this condition.

### Participants

The feasibility study will aim to recruit 30 participants from two NHS hospital sites in the West of England. Recruitment will take place between September 2019 and September 2020. As this is a feasibility trial, it is not appropriate to perform formal power calculations. Instead a sample size of 30 was chosen based on the practicalities of conducting an early-stage intervention trial within the scope of a Ph.D. project. This sample size will also allow for the estimation of parameters for potential primary outcomes in a definitive trial, helping to inform future sample size calculations [23].

Eligible participants will provide written informed consent, taken by a member of the site-based research team. Participants will be deemed eligible if they are aged ≥ 18 years with histologically confirmed stage 2/3 colorectal cancer, are due to undergo treatment with adjuvant CAPOX chemotherapy, have an ECOG performance status ≤ 2 and are able to provide written informed consent. People will be excluded if they have a clinical diagnosis of cachexia or diabetes, a body mass index (BMI) ≤ 18.5 kg/m^2^, history of an eating disorder, or drug/alcohol abuse are participating in another study that may affect the outcomes of this feasibility trial or are unable to speak/understand English.

Treatment discontinuation will be at the discretion of the principal investigator or other attending clinician or the participant themselves. All participants who withdraw from the trial intervention will be followed up as per the follow-up schedule until trial completion. The exception to this will be if a participant explicitly withdraws consent for any further trial follow-up.

### Randomisation and allocation

Randomisation will be completed in a 1:1 ratio using random permuted blocks, by a secure online randomisation system.

Blinding of the participant to the outcome of randomisation will not be possible, due to the nature of this intervention. However, outcome assessors of the markers of cellular metabolism will remain blind to participants’ random allocation.

### Trial arms

Following randomisation, participants will be given verbal and written instructions related to their allocated trial arm by a member of the trial team. These instructions will detail how and when to complete the self-reported data and, for the intervention arm, how and when to implement the fast.

Participants randomised to the intervention arm will undertake a 36-h water-only fast, immediately prior to chemotherapy administration for the first 3 cycles. A decrease in IGF-I levels in response to a short-term fast in humans is seen within 36–120 h of fasting [3]. As previous research has suggested that longer fasts may be subject to poorer adherence [10], we have selected a 36-h fast prior to each of the first 3 cycles of chemotherapy. This aims to find the balance between implementing a fast that is long enough to alter metabolic pathways whilst keeping participant burden to a minimum.

Participants randomised to the control arm of the trial will receive standard dietary guidance/advice as per local standard practice. This may include verbal or written information on the diet and effects of chemotherapy on appetite.

### Study schedule

The SPIRIT figure is shown in Table 1, which outlines the study schedule and the SPIRIT checklist has been provided as an Additional file 1. Baseline assessments will take place when the patient attends their screening visit, following successful screening and enrolment. The intervention will be implemented for the first 3 cycles of their chemotherapy schedule. Follow-up data will be collected on days 1, 3, and 7 of each cycle. Adverse events and concomitant medication will be monitored throughout trial participation. At the end of their trial participation, each participant will resume their usual care pathway.

**Table 1:**
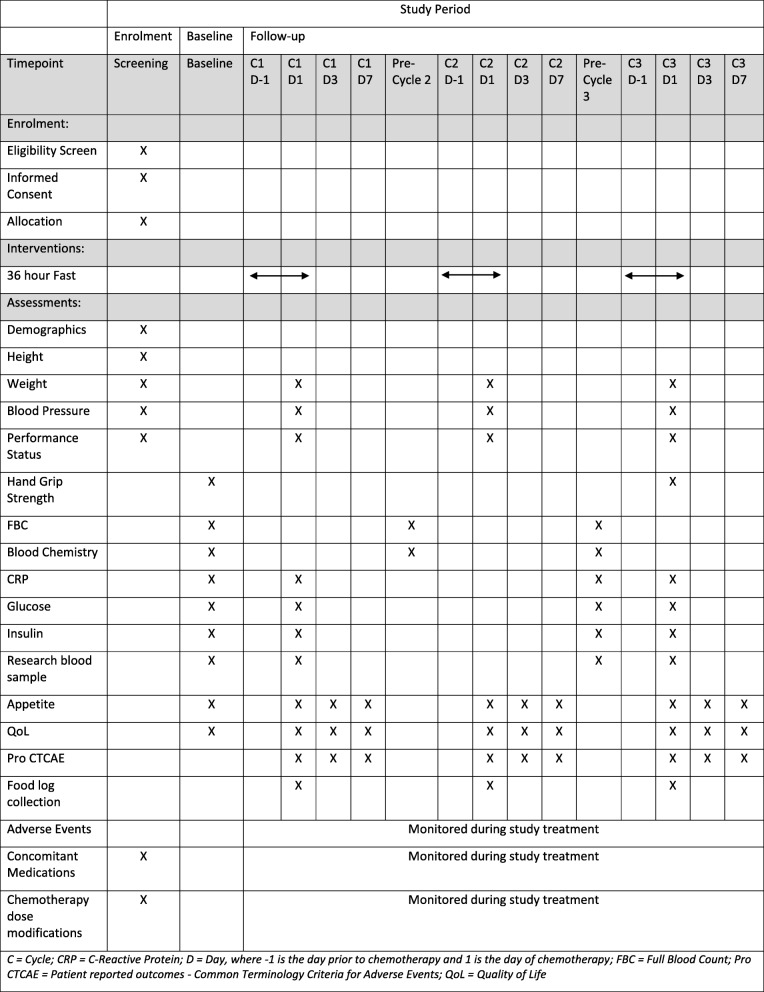
Trial schedule

C = cycle; CRP = C-reactive protein; D = Dday, where −1 is the day prior to chemotherapy and 1 is the day of chemotherapy; FBC = full-blood count; Pro CTCAE = Patient reported outcomes-Common Terminology Criteria for Adverse Events; QoL = quality of life

The end of the trial will be defined as the completion of data queries, and sample and data analysis.

### Embedded qualitative study

Participants will be invited to take part in one semi-structured qualitative interview once they have completed the trial. These will be conducted face-to-face in the clinic. However, telephone interviews will be arranged where face-to-face interviews are not possible.

Interviews will follow a topic guide, which covers topics such as dietary guidance received, experience of randomisation, tolerability of the intervention/experience of taking part as a “control”, and experience of the data collection methods. It will also discuss any barriers or enabling factors that participants experienced in adhering to the fast, with the view to informing future trials of fasting interventions. However, an open discussion will be promoted, and the topic guide will be continually reviewed throughout the interview process to ensure it covers any emerging topics of interest.

### Biological specimens

All human tissue samples will be collected, used, and stored in accordance with the Human Tissue Act 2004. FBC and blood chemistry data will be collected from routine samples analysed in NHS labs as per standard practice. This data will be collected from participant medical records by a member of their clinical care team or research team member based on their site.

As per the trial schedule (Table 1), additional baseline and follow-up samples obtained immediately prior to chemotherapy administration at cycles 1 and 3 will be analysed within the NHS labs for glucose, insulin, and CRP.

Additional blood samples will be collected at baseline and chemotherapy day 1 visits for cycles 1 and 3 for analysis of IGF-I, IGF-II, IGFBP-2, and IGFBP-3. Following collection, serum tubes will be inverted 8–10 times, then allowed to sit at room temperature, in the dark (e.g. within an envelope) for 30–60 min to clot. Samples will be centrifuged at 2500 rpm for 15 min, and the serum pipetted off the top for storage. This will be aliquoted into 1.5 ml Eppendorf tubes (1ml per tube) for freezing (at − 80 °C) and storage at the NHS site until transfer to the University of Bristol laboratories for analysis at the end of the trial.

### Quantitative data analysis

Rates and confidence intervals will be reported for the primary outcomes. Baseline characteristics in each trial arm will be reported. Secondary outcome data will be summarised using means (standard deviations) or medians (inter-quartile ranges) as appropriate for continuous variables, and frequencies with percentages (n, %) for categorical variables, to inform outcomes of interest and sample size calculations in future trials. All quantitative data will be analysed in STATA 15 [24].

### Qualitative data analysis

Audio recordings of the interviews will be transcribed verbatim by a sponsor approved transcription company. The anonymised data will be analysed using the framework method, a form of thematic analysis [25]. A coding index, based on the interview topic guide, will be used to sort the data into themes. An inductive approach to analysis will be used, allowing emergent themes to alter the coding as the analysis progresses. Coding will be completed by a single researcher, then reviewed by a second researcher to ensure both consistency of coding and grounding in the original data. Any inconsistencies in themes or coding will be discussed and resolved between the two researchers. This process will take place in parallel with the data collection to allow any emerging themes to be further explored in subsequent interviews. A framework matrix will then be created using participants’ responses to each theme. Qualitative data analysis will be assisted by Nvivo 10 software [26].

## Discussion

This is the first study of short-term fasting in people due to undergo chemotherapy for colorectal cancer. Fasting during the treatment of cancer is an area of growing research interest, due to the findings of pre-clinical research in animal, cell line, and yeast models. This research has identified fasting as a potential tool for reducing the toxicities associated with cancer treatments such as chemotherapy and radiotherapy, as well as potentially improving tumour response and survival rates. However, although some studies of fasting during chemotherapy in pre-clinical models have shown promising results, it is unclear how well these findings translate to humans.

SWiFT will allow us to test the feasibility of conducting a fasting intervention in people undergoing chemotherapy for colorectal cancer, a population previously unstudied in this area. The embedded interview study will also allow us to qualitatively assess patient experiences of the intervention and data collection methods used in the trial. We will use validated questionnaires to collect data on treatment side effects experienced by trial participants. Although not powered to determine effects of the fast on these treatment toxicities, collecting data on the secondary outcomes such as chemotherapy side effects and markers of cellular metabolism, will help to inform sample size calculations in future trials.

## Supplementary information

**Additional file 1.** SWiFT SPIRIT checklist.

## Data Availability

The anonymised datasets generated by the current study will be stored for up to 20 years on the University of Bristol’s secure online Research Data Storage Facility. Data will only be accessible to members of the study research team but may also be made available on reasonable request by contacting the corresponding author.

## Abbreviations

BMI: Body mass index
BMR: Basal metabolic rate
CAPOX: Capecitabine oxaliplatin chemotherapy
CRP: C-reactive protein
CT: Computerised tomography
CTCAE: Common Terminology Criteria for Adverse Events
DSR: Differential stress resistance
EQ-5D-5L: Euroqol, 5 dimensions, 5 levels: quality of life measure
FBC: Full-blood count
IGF: Insulin-like growth factor
IGFBP: Insulin-like growth factor-binding protein
NHS: National Health Service
Pro-CTCAE: Patient-reported outcomes-Common Terminology Criteria for Adverse Events
QoL: Quality of life
RCT: Randomised controlled trial
SWiFT: Short-term, water only, fasting trial

## References

[1] Longo VD, Fontana L (2010). Calorie restriction and cancer prevention: metabolic and molecular mechanisms. Trends in Pharmacological Sciences.

[2] Fontana L, Partridge L, Longo VD (2010). Extending Healthy Life Span—From Yeast to Humans. Science.

[3] Lee C, Longo VD (2011). Fasting vs dietary restriction in cellular protection and cancer treatment: from model organisms to patients. Oncogene.

[4] Hanahan D, Weinberg RA. Hallmarks of Cancer: The Next Generation. Cell. 144(5):646–74. 10.1016/j.cell.2011.02.013 21376230

[5] Raffaghello L (2008). Starvation-dependent differential stress resistance protects normal but not cancer cells against high-dose chemotherapy. Proceedings of the National Academy of Sciences.

[6] van Niekerk G, Hattingh SM, Engelbrecht A-M (2016). Enhanced Therapeutic Efficacy in Cancer Patients by Short-term Fasting: The Autophagy Connection. Frontiers in Oncology.

[7] O’Flanagan CH (2017). When less may be more: calorie restriction and response to cancer therapy. BMC Medicine.

[8] Sun L (2017). Effect of fasting therapy in chemotherapy-protection and tumorsuppression: a systematic review. Translational Cancer Research.

[9] de Groot S (2015). The effects of short-term fasting on tolerance to (neo) adjuvant chemotherapy in HER2-negative breast cancer patients: a randomized pilot study. BMC Cancer.

[10] Dorff TB (2016). Safety and feasibility of fasting in combination with platinum-based chemotherapy. BMC Cancer.

[11] Poston GJ (2011). Diagnosis and management of colorectal cancer: summary of NICE guidance. BMJ.

[12] Zeuli M (2001). Capecitabine and oxaliplatin in advanced colorectal cancer: a dose-finding study. Annals of Oncology.

[13] Schmoll H-J (2015). Capecitabine Plus Oxaliplatin Compared With Fluorouracil/Folinic Acid As Adjuvant Therapy for Stage III Colon Cancer: Final Results of the NO16968 Randomized Controlled Phase III Trial. Journal of Clinical Oncology.

[14] Henry CJK (2005). Basal metabolic rate studies in humans: measurement and development of new equations. Public Health Nutrition.

[15] National Cancer Institute. Patient-Reported Outcomes version of the Common Terminology Criteria for Adverse Events (PRO-CTCAE™). 2018 [cited 2018 26 Mar]; Available from: https://healthcaredelivery.cancer.gov/pro-ctcae/.

[16] The EuroQol Group (1990). EuroQol-a new facility for the measurement of health-related quality of life. Health Policy.

[17] National Institute of Health. *Common Terminology Criteria for Adverse Events (CTCAE)*. 2009 [cited 2017 13 Nov]; Available from: https://evs.nci.nih.gov/ftp1/CTCAE/CTCAE_4.03_2010-06-14_QuickReference_5x7.pdf

[18] Blundell J (2010). Appetite control: methodoligical aspects of evaluation of foods. Obes rev.

[19] Boltong, A., et al., A prospective cohort study of the effects of adjuvant breast cancer chemotherapy on taste function, food liking, appetite and associated nutritional outcomes*.* PloS one, 2014. 9(7): p. e103512-e103512. 10.1371/journal.pone.0103512 PMC4117507 25078776

[20] Vega MCMD, Laviano A, Pimentel GD (2016). Sarcopenia and chemotherapy-mediated toxicity. Einstein.

[21] Slice-O-matic; Tomovision, Montreal, QC, Canada.

[22] Bahat G (2016). *Cut-off points to identify sarcopenia according to European Working Group on Sarcopenia in Older People (EWGSOP) definition*. Clinical Nutrition.

[23] Lancaster GA, Dodd S, Williamson PR (2004). Design and analysis of pilot studies: recommendations for good practice. J Eval Clin Pract..

[24] StataCorp., Stat Statistical Software: Release 15. 2017, StataCorp LLC: College Station, TX.

[25] Ritchie JSE (2002). The qualitative researcher's companion.

[26] QSR International Pty Ltd (2012). NVivo qualitative data analysis Software.

